# Hantaviruses and Hantavirus Pulmonary Syndrome, Maranhão, Brazil

**DOI:** 10.3201/eid1612.100418

**Published:** 2010-12

**Authors:** Elizabeth S. Travassos da Rosa, Elba R. Sampaio de Lemos, Daniele B. de Almeida Medeiros, Darlene B. Simith, Armando de Souza Pereira, Mauro R. Elkhoury, Wellington S. Mendes, José R.B. Vidigal, Renata C. de Oliveira, Paulo S. D’Andrea, Cibele R. Bonvícino, Ana C.R. Cruz, Márcio R.T. Nunes, Pedro F. da Costa Vasconcelos

**Affiliations:** Author affiliations: Evandro Chagas Institute, Ananindeua, Brazil (E.S. Travassos da Rosa, D.B. de Almeida Medeiros, D.B. Simith, A. de Souza Pereira, A.C.R. Cruz, M.R.T. Nunes, P.F. da Costa Vasconcelos);; Oswaldo Cruz Institute, Rio de Janeiro, Brazil (E.R. Sampaio de Lemos, R.C. de Oliveira, P.S. D’Andrea);; Pan American Health Organization, Brasília, Brazil (M.R. Elkhoury);; Federal University of Maranhão, São Luís, Brazil (W.S. Mendes);; State Health Secretariat, São Luís (J.R.B. Vidigal);; National Institute of Cancer, Rio de Janeiro (C.R. Bonvicino);; Pará State University, Belém, Brazil (A.C.R. Cruz, P.F. da Costa Vasconcelos)

**Keywords:** Hanvavirus pulmonary syndrome, hantavirus, Anajatuba virus, Oligoryzomys fornesi, Brazil, viruses, zoonoses, dispatch

## Abstract

To confirm circulation of Anajatuba virus in Maranhão, Brazil, we conducted a serologic survey (immunoglobulin G ELISA) and phylogenetic studies (nucleocapsid gene sequences) of hantaviruses from wild rodents and persons with hantavirus pulmonary syndrome. This virus is transmitted by *Oligoryzomys fornesi* rodents and is responsible for hantavirus pulmonary syndrome in this region.

Hantaviruses (family *Bunyaviridae*, genus *Hantavirus*) cause a viral zoonosis transmitted by rodents belonging to the families Muridae and Cricetidae. Each hantavirus is predominantly associated with a specific rodent species in a specific geographic region. However, infection of other rodent species can occur as a spillover phenomenon (*1*).

Hantavirus disease has 2 recognized clinical forms, hemorrhagic fever with renal syndrome and hantavirus pulmonary syndrome (HPS) (*2*). The respiratory form of the disease was described in June 1993 during an epidemic of severe respiratory disease caused by Sin Nombre virus in the United States (*3*). A few months later, 3 HPS cases were identified in 3 siblings in Juquitiba, São Paulo State, Brazil (*4*). During 1993–2009, a total of 1,246 HPS cases (264 in the Amazon region) were reported in Brazil, and new hantaviruses were identified (Juquitiba virus, Castelo dos Sonhos virus, Araraquara virus, Anajatuba virus, and Rio Mearim (*5*).

During 2003–2005, an ecoepidemiologic study was conducted in the municipality of Anajatuba, Maranhão, Brazil, to identify reservoirs of hantaviruses after identification of 3 HPS cases (*6*). Two new hantaviruses, Anajatuba virus and Rio Mearim virus, were isolated from *Oligoryzomys fornesi* (rice rat) rodents and *Holochilus sciureus* (marsh rat) rodents, respectively, and genetically characterized (*5*). To confirm circulation of Anajatuba virus in Maranhão, Brazil, we conducted a serologic survey (immunoglobulin [Ig] G ELISA) and phylogenetic studies (nucleocapsid gene sequences) of hantaviruses obtained from wild rodents and persons with HPS.

## The Study

**Figure 1:**
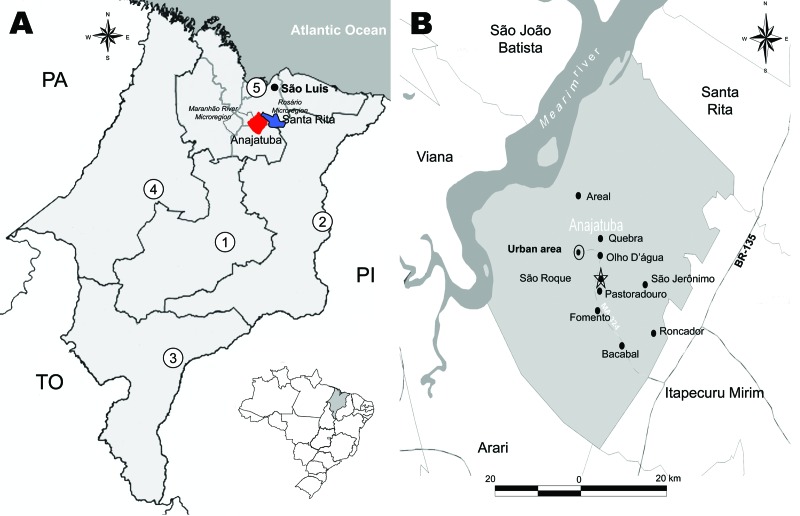
A) Regions of Anajatuba (red) (Maranhão River Microregion) and Santa Rita (blue) (Rosario Microregion), Maranhão, Brazil, where hantavirus pulmonary syndrome (HPS) cases were found. PA, Para; TO, Tocantins; PI, Piaui; 1, central region; 2, eastern region; 3, southern region; 4, western region; 5, northern region. B) Towns in Anajatuba where a serologic survey for HPS in humans was performed. Dotted oval, São Roque; star, rodent capture location; ovals, locations where HPS cases were found.

Anajatuba (3°16′S, 44°37′W; population 23,907) and Santa Rita (3°9′S, 44°20′W; population 31,033) (www.ibge.gov.br), are located in the western floodplain of the Maranhão River in Maranhão State, Brazil (Figure 1, panel A). The region has chains of lakes with extensive swamps and flooded fields, forest areas, and rice fields extending from the outskirts of the urban area. The climate is tropical and humid (average temperature range 26°C–28°C), and the rainy season is during January–July (*5**,**6*).

Data for 5 cases of HPS in men (age range 25–30 years, 3 from Anajatuba and 2 from Santa Rita) are shown in Table 1. In a cross-sectional serologic survey in residents of Anajatuba, 293 serum samples (8.1% of the population studied and 1.2% of the total population of the municipality) were obtained; 153 (52%) residents were women. Fifty-four samples were obtained from urban residents, and 239 samples were obtained from rural residents. All samples were tested by using an ELISA to detect IgM and IgG as described (*7*).

**Table 1:** Characteristics of 5 human case-patients with hantavirus pulmonary syndrome and 3 rodents infected with hantavirus, Maranhão, Brazil*

Sample origin†	Municipality/town	Patient age, y	Sample collection date	Symptom duration, d	Clinical outcome	ELISA results		GenBank accession no.
						IgG	IgM	
Human								
Be H 666379‡	Anajatuba/São Roque	24	2003 Mar 25	1	Died	Neg	Pos	HM238889
Be H 668281	Santa Rita/Conceição	21	2003 May 14	6	Recovered	Pos	Pos	–
Be H 670957‡	Anajatuba/Fomento	24	2003 Jul 22	4	Recovered	Pos	Pos	HM238890
Be H 672862‡	Santa Rita/NA	39	2003 Oct 21	10	Recovered	Neg	Pos	HM238885
Be H 708080	Anajatuba/Roncador	28	2006 Jun 12	NA	Died	Neg	Pos	–
Rodent				Species				
BeAN669104‡	Anajatuba/São Roque	NA	2003 May 27	*Necromys lasiurus*		Pos	ND	HM238886
BeAN690936‡	Anajatuba/São Roque	NA	2005 May 18	*Oligoryzomys fornesi*		Pos	ND	HM238887
BeAN690985‡	Anajatuba/São Roque	NA	2005 May23	*O*. *fornesi*		Pos	ND	HM238888

*Ig, immunoglobulin; Neg, negative; Pos, positive; NA, not available; ND, not done. †Serum samples were obtained from humans (males), and lung tissue samples were obtained from rodents (males). ‡Amplicons for these samples were sequenced.

The main findings of the serologic study are shown in Table 2. A male:female ratio of 2:1 was observed in urban and rural areas. Factors investigated for increasing risk for exposure to hantaviruses included living near rice paddies; engaging in farming or fishing; having wild rodents around the household; having contact with wild rodents in the workplace, school, or domestic surroundings; and storing rice in the household.

**Table 2:** Testing for hantavirus among residents of urban and rural areas of Anajatuba, Maranhão, Brazil, May 2005*

Zone	Town	Total population	No. (%) persons sampled	No. (%) persons positive†
Urban	Limirique and Porção do Junco (neighborhood)	1,059	54 (5.09)	9 (16.7)
Rural	Areal	375	42 (11.2)	3 (7.1)
	Bacabal	790	57 (7.2)	7 (12.3)
	Olho d’água	193	51 (26.4)	5 (9.8)
	Quebra	584	38 (6.5)	5 (13.2)
	São Roque and Pastoradouro	634	51 (8.0)	3 (5.9)
Rural zone total		2,576	239 (9.3)	23 (9.6)
Total		3,635	293 (8.1)	32 (10.9)

*Data were provided by the Health Municipal Secretary of the Anajatuba Municipality, 2005. †By immunoglobulin G testing.

In May 2003 and May 2005, two rodent captures approved by the Instituto Brasileiro do Meio Ambiente e dos Recursos Naturais Renováveis/Instituto Chico Mendes de Conservação da Biodiversidade were conducted in São Roque, Anajatuba (Figure 1, panel B). Trapping was conducted <50 m from residences of 3 deceased HPS case-patients in accordance with accepted rodent capture and handling procedures and standard biosafety protocols for anesthetizing and killing rodents, and biometric analysis was conducted (*8*). Fragments of liver, lung, spleen, heart, and kidney were obtained. Taxonomic identification was performed according to procedures of Bonvincino and Moreira (*9*).

Biologic samples (blood and viscera fragments) were obtained from 216 captured rodents: 96 (44%) captured in 2003 and 120 (56%) captured in 2005. The most common species captured in 2003 were *Necromys lasiurus* rodents (n = 62, 64%) and *Akodon* sp. rodents (n = 27, 28%). The most common species captured in 2005 were *N. lasiurus* rodents (n = 105, 87%) and *O*. *fornesi* rodents (n = 2, 2%); the remaining rodents were from other genera.

Blood samples collected from wild rodents were also tested by using an IgG ELISA (*10*). IgG against hantavirus was detected in 2 (100%) of 2 *O. fornesi* rodents captured in 2005 and 6 (4%) of 167 *N. lasiurus* rodents (3 of 62 captured in 2003 and 3 of 105 captured in 2005) (Table 1).

Virus RNA was extracted from IgM-positive human serum or blood samples and lung fragments from IgG-positive rodents by using the QIAamp Viral RNA Mini Kit (QIAGEN, Valencia, CA, USA) according to the manufacturer’s instructions. Nested reverse transcription–PCR and hemi-nested reverse transcription–PCR were used for amplification of partial nucleocapsid gene sequences from human and rodent samples, respectively, by using primers described (*11*). Purified amplicons were obtained by using the GFX PCR DNA and Gel Band Purification Kit (Healthcare, Little Chalfont, UK) and sequenced. Amplicons (434 bp) generated from HPS cases in humans (2 from Anajatuba and 1 from Santa Rita) and from 3 of 8 lung samples from hantavirus IgG-positive rodents were sequenced (Table 1).

Phylogenetic trees were constructed by using neighbor-joining, maximum-parsimony, maximum-likelihood, and Bayesian methods implemented in PAUP 4.0b.10 (*12*), PHYML (*13*), and BEAST (*14*). Modeltest version 3.6 (*15*) was used to determine the best nucleotide substitution model based on Akaike information criteria. Analyses were conducted by using confidence values estimated from mean nucleotide divergence obtained for different Old World and New World hantavirus sequences by using MEGA version 3.0 software (www.megasoftware.net) Estimated values were <45%, <25%, 22%, and 15% and were used for grouping viruses in clusters, clades, subclades, and species, respectively.

**Figure 2:**
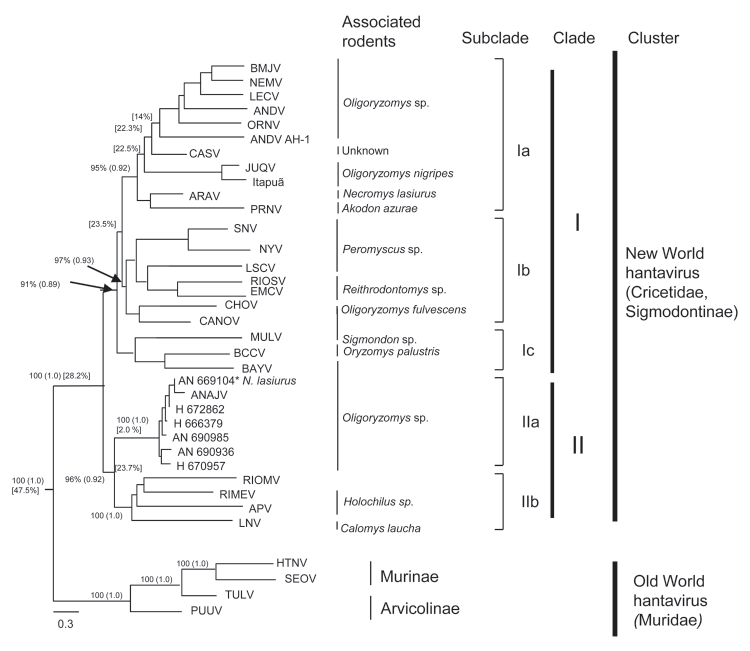
Phylogenetic analysis of partial small RNA segments of hantaviruses, Maranhão, Brazil, by using maximum-likelihood and Bayesian methods. Bayesian and bootstrap values (in parentheses) are shown over each main tree node. Values in brackets indicate mean divergence between groups. Arrows indicate exact position of these 2 values. Scale bar indicates nucleotide sequence divergence. BMJV, Bermejo virus; NEMV, Neembuco virus; LECV, Lechiguanas virus; ANDV, Andes virus; ORNV, Oran virus; CASV, Castelo dos Sonhos virus; JUQV, Juquitiba-Araucaria virus; ARAV, Araraquara virus; PRNV, Pergamino; SNV, Sin Nombre virus; NYV, New York virus; LSCV, Limestone Canyon virus; RIOSV, Rio Segundo virus; ECMV, El Moro Canyon virus; CHOV, Choclo virus; CANOV, Cano Delgadito virus; MULV, Muleshoe virus; BCCV, Black Creek Canal virus; BAYV, Bayou virus; ANAJV, Anajatuba virus; RIOMV, Rio Mamoré virus; RIMEV, Rio Mearim virus; APV, Alto Paraguay virus; LNV, Laguna Negra virus; HTNV, Hantaan virus; SEOV, Seoul virus; TULV, Tula virus; PPUV, Puumala virus.

All phylogenetic methods showed similar topologies, and the ML maximum-likelihood construction was selected for representing the final tree. Bootstrap and Bayesian posterior probability values are shown in Figure 2.

Two major clusters were observed (New World and Old World hantavirus groups) and had a genetic distance of 28.2% (inclusion value 25%). The New World group was divided into clades I and II. Clade I was divided into 3 subclades (genetic divergence 23.5%), Ia, Ib, and Ic. Clade II was divided into 2 subclades (genetic divergence 23.7%), IIa and IIb. The strains used in this study were closely related to Anajatuba virus and were included in the IIa subclade (genetic divergence 2%) (Technical Appendix ).

Nucleotide and amino acid homology between Anajatuba virus (*5*) and the strains isolated in this study in Maranhão were 98.3% and 100%, respectively. These strains were included in a group related to rodents belonging to the genus *Oligoryzomys*, although sample Be AN 669104 was obtained from an *N. lasiurus* rodent, which suggests spillover transmission between rodent species.

## Conclusions

We showed that Anajatuba virus is responsible for human HPS cases and that *O. fornesi* rodents are its likely reservoir. Anajatuba virus infections of *N. lasiurus* were spillover infections. Human hantavirus infections are common among persons in the Baixada Maranhense region, but cases of HPS are rare. However, educational and health surveillance programs are needed to prevent hantavirus transmission.

## Supplementary Material

Technical Appendix Distribution of hantavirus groups and subgroups in the Western Hemisphere.
