# Supplementary material for: Hantaviruses and Hantavirus Pulmonary Syndrome, Maranhão, Brazil
Source: Emerg Infect Dis. 2010 Dec;16(12):1952–5. doi: 10.3201/eid1612.100418 (PMC3294554; doi:10.3201/eid1612.100418)
Supplement: Technical Appendix — Distribution of hantavirus groups and subgroups in the Western Hemisphere. [file 10-0418-Techapp_1p.pdf]

Article DOI: 10.3201/eid1612.100418

Hantaviruses and Hantavirus Pulmonary Syndrome, Maranhão, Brazil

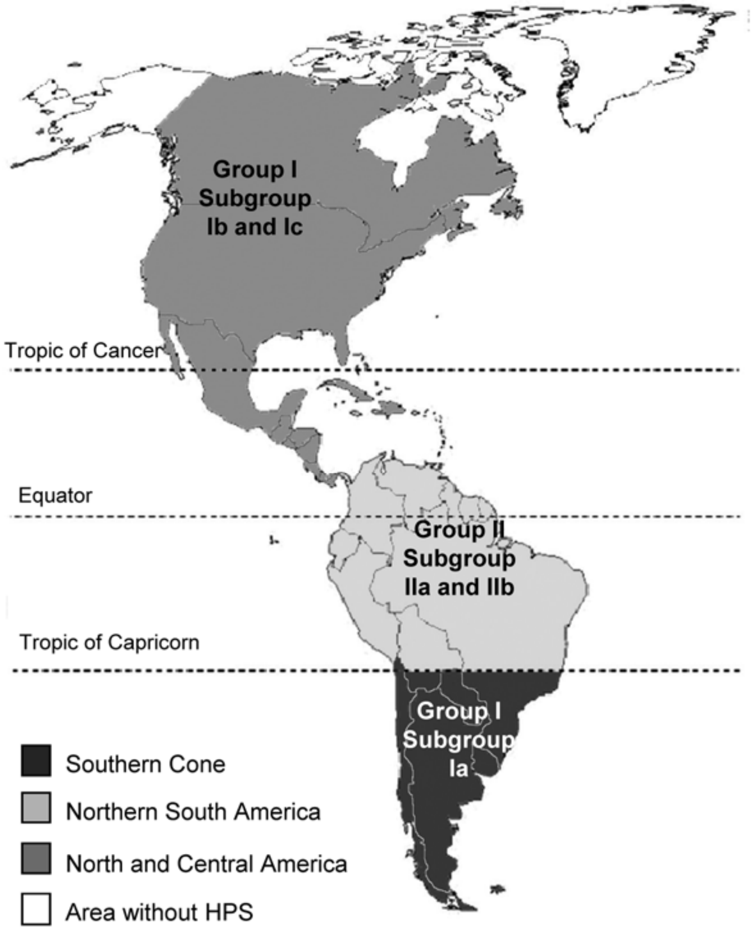

Technical Appendix Figure. Distribution of hantavirus groups and subgroups in the Western Hemisphere. HPS, hantavirus pulmonary syndrome.
